# Reinforced scan: a reinforcement learning enabled optimal laser scan path planning in laser powder bed fusion additive manufacturing

**DOI:** 10.1007/s00170-025-17144-9

**Published:** 2026-01-24

**Authors:** Chaoran Dou, Jihoon Chung, Raghav Gnanasambandam, Yuhao Wu, Jianzhi Li, Zhenyu James Kong

**Affiliations:** 1 Department of Industrial and Systems Engineering, University of Missouri, Columbia, MO 65211 USA; 2 Department of Industrial Engineering, Hanyang University, Seoul, Korea; 3 Department of Industrial and Manufacturing Engineering, FAMU-FSU College of Engineering, Tallahassee, FL 32310 USA; 4 Department of Manufacturing and Industrial Engineering, The University of Texas Rio Grande Valley, Edinburg, TX 78539 USA; 5 Grado Department of Industrial and Systems Engineering, Virginia Tech, Blacksburg, VA 24061 USA

**Keywords:** Reinforcement learning, Laser powder bed fusion, Scan strategy, Defect mitigation, Optimization

## Abstract

Additive Manufacturing is an innovative technology that fabricates parts layer by layer. However, in Laser Powder Bed Fusion (LPBF), printed metal parts often exhibit residual stresses, deformations, and other defects due to non-uniform temperature distribution during the printing process. To mitigate these issues, an optimized scan sequence within each layer can improve thermal uniformity. Traditional optimization methods, which rely on domain knowledge and employ trial-and-error or heuristic approaches, often fail to achieve optimal solutions due to the complex nature of the problem. One major challenge in improving scan strategies lies in the vast search space required to optimize the scan sequence for individual scan tracks within each layer, making it difficult to identify the best solution. To overcome this challenge, this work proposes an innovative scan strategy, Reinforced Scan, that leverages reinforcement learning to intelligently determine the optimal scan sequence. The method introduces a novel reward function that accounts not only for temperature variance but also for the spatial uniformity of the temperature field. By structuring the optimization problem into multiple hierarchical levels, the approach significantly reduces computational demand and enhances the manageability of the optimization process. The effectiveness of the proposed Reinforced Scan is validated through Netfabb™ Local Simulation and real-world laser scanning experiments on a Ti-6Al-4V thin plate. Its performance is compared against conventional heuristic scan sequences. Both simulation and experimental results demonstrate that Reinforced Scan achieves superior outcomes, notably reducing residual stress compared to traditional methods.

## Introduction

Additive Manufacturing (AM) fabricates 3D parts from digital models by progressively adding material layer by layer [1]. Among all AM technologies, Laser Powder Bed Fusion (LPBF) is one of the most popular approaches to additive manufacturing metal parts [2]. It is applied in various industries because of its advantage in manufacturing parts with detailed features and dense structure. LPBF uses a laser as the heat source to melt metal powder during the layer-wise manufacturing process. The laser irradiates a thin layer of metal powder, inducing rapid melting and subsequent formation of a molten pool; within this pool, intense thermal gradients drive fluid flow via Marangoni convection and recoil pressure from vaporization [3–7]. As the melt pool solidifies, the high cooling rates and steep temperature gradients resulting from poor thermal uniformity promote early nucleation and directional grain growth, often resulting in columnar microstructures and associated residual stresses and defects, such as deformation and cracking [3, 8]. Common solutions for residual stress are reducing thermal gradients during the build or post-processing heat treatment [9–12]. However, the post-processing heat treatment has a high time cost and cannot solve in-process deformation and cracking [10, 11]. Therefore, minimizing the residual stress by uniforming the temperature distribution during the process is preferred [10]. An effective approach is to optimize the scan strategy, especially the scan sequence in a scan pattern.

**Fig. 1:**
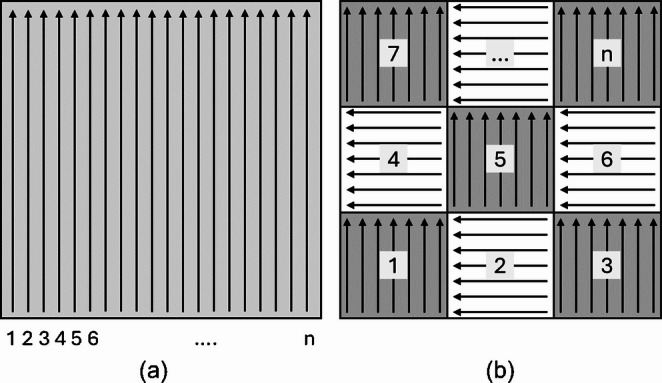
Two commonly used scan patterns in LPBF are (**a**) stripe pattern and (**b**) chessboard pattern. The chessboard pattern consists of stripe patterns that divide the scan space into islands

The two most common scan patterns in LPBF are stripes and chessboard [10], as shown in Fig.1: (a) stripe pattern and (b) chessboard pattern. The stripe pattern consists of linear scan lines, while the chessboard pattern divides the scan area into multiple islands (namely, sub-regions). The scan sequence can be defined as the order of those lines in a stripe pattern or islands in a chessboard pattern scanned by laser or electron beam. In the two patterns, if the lines or islands are indexed as 1, 2, 3, …, n, the scan sequence can be defined as an ordered list of these indices, determining the specific order in which the lines or islands are scanned. For example, a sequential scan means that the scanning progresses in order from one end to the other, following the ordered list [1, 2, 3, 4, …, n]. The optimization of the scan sequence in LPBF involves determining the most effective sequence for executing predefined scan actions, such as lines in stripe patterns or islands in chessboard patterns. The objective of this optimization is to minimize residual stress in the printed parts [10, 13]. This optimization process is critical because sub-optimal scan sequences will lead to non-uniform temperature distribution, large thermal gradients, and high cooling rates. These thermal inconsistencies result in defects such as excessive residual stress and deformation, which compromise the structural integrity and dimensional accuracy of the final additively manufactured products. By optimizing the scan sequence, the thermal uniformity can be improved, and the residual stress can be minimized.

Recently, researchers have developed new approaches for optimizing scan sequences and printing patterns to enhance the quality of LPBF. Kruth et al. [14] introduced the Least Heat Influence (LHI) approach, which strategically arranges the next scan as far away as possible from the previous scan, and it demonstrated effectively reduced residual stresses than other methods [15]. Malekipour et al. [16] proposed a genetic algorithm-based method named Genetic Algorithm Maximum Path (GAMP), which utilizes a genetic algorithm to maximize the path connecting the centers of all islands. They also proposed another genetic algorithm-based method named novel online thermography and closed-loop hybrid control (NOTCH) [17]. However, the specific problem can only be solved with a small number of islands. Ramos et al. [18] proposed the intermittent laser path to avoid scanning adjacent areas consecutively. This approach utilizes a geometry-based formula to reduce thermal-induced deformation. Based on the nearest neighbor method, Ramani et al. proposed the SmartScan [10]. In SmartScan, the thermal model exhaustively searches all possible next scan positions and selects the one that results in the minimum temperature variance. However, SmartScan tends to focus on optimizing each scan step independently, which may limit its ability to find the best overall scanning sequence. Qin et al. [19] proposed a deep reinforcement learning (DRL)-based framework for generating toolpaths in islands to achieve thermal uniformity and minimize residual stress. The DRL-based algorithm optimizes the movement of the laser beam to avoid regions of high energy density, leading to more uniform temperature fields during printing. However, this work did not consider the heat transfer over time and the scanning sequence of islands.

The discussion above highlights a critical gap in the optimization of scan strategies in LPBF additive manufacturing. Existing studies either do not consider the dependency between scan steps or only optimize a small portion of the entire scan area, which limits their effectiveness in further reducing residual stress and deformation in LPBF parts. This limitation stems from the combinatorial explosion of the scan sequence search space. The total number of possible scan sequences increases factorially with the number of scans, resulting in an extremely large search space. Consequently, exhaustively searching this space becomes computationally intractable. Moreover, conducting a large number of simulations or experiments to evaluate thermal distribution for each candidate sequence is prohibitively expensive. Therefore, the traditional design of experiments (DOE) approaches are not viable for exploring the entire design space.

To tackle this challenge, this paper presents a Reinforcement Learning (RL) framework tailored to the scan sequence optimization problem [20, 21]. In particular, Deep Q-Learning (DQL) [22–24] is utilized to efficiently search for an optimal scan sequence that minimizes thermal-related defects.

This proposed method leverages physics-based simulation models together with RL models to efficiently determine the optimal scan sequence for LPBF. By utilizing an innovative reward function, the Reinforced Scan reduces the residual stress and deformation by improving the uniformity of temperature distribution during the AM process. Reinforced Scan stands out for two main features: First, the Reinforced Scan method can reduce the computational power required for optimizing scan sequence in LPBF. Second, the Reinforced Scan is not focused on individual steps, which can generate a holistic result better than traditional heuristic methods.

The rest of this paper is structured as follows: Sect. 2 introduces the Reinforced Scan, including optimization using reinforcement learning, hierarchical optimization, and a customized reward function. Section 3 presents case studies comparing the Reinforced Scan with traditional methods. Section 4 provides the Conclusion, summarizing the entire work.

## Reinforced Scan: a reinforcement learning-enabled scan strategy

This section introduces Reinforced Scan, an RL-based optimization method developed to improve the scan sequence in LPBF. By modeling scan sequence selection as a sequential decision-making process, this method employs DQL to intelligently explore the large decision space of possible scan orders.

### The overall methodology based on deep Q-learning

DQL is an advanced RL algorithm that merges traditional Q-Learning, a model-free RL method, with deep neural networks [19, 25]. Traditional Q-Learning is a value-based RL algorithm that aims to determine the optimal action-selection policy for any given state by iteratively updating a Q-value table,*Q* (*s*,* a*), which estimates the expected return (i.e., accumulated rewards) of taking action 𝑎 in state s [24, 26]. In the context of scan sequence optimization for LPBF, the state is defined as the current temperature distribution, the action corresponds to selecting the next scan location, and the reward quantifies the thermal uniformity of the resulting temperature field. The Q-values are updated using the Temporal Difference (TD) learning method [27]:

1 $$\:Q(s,a)\leftarrow\:(1-\alpha\:)Q(s,a)+\alpha\:[r+\gamma\:{\mathrm{m}\mathrm{a}\mathrm{x}}_{a{\prime\:}}\text{}Q(s{\prime\:},a{\prime\:}\left)\right]$$

where Q (*s*,* a*) is the current Q-value for taking action *a* in state *s*, $\:\alpha\:$ is the learning rate, *r* is the received reward after taking action a, $\:\gamma\:$ is the discount factor reflecting the importance of future rewards, $\:s{\prime\:}$ is the next state resulting from taking action *a* in state *s*, and $\:{\mathrm{m}\mathrm{a}\mathrm{x}}_{a{\prime\:}}\text{}Q(s{\prime\:},a{\prime\:})$ is the maximum Q-value estimated for the next state $\:s{\prime\:}$ over all potential actions $\:a{\prime\:}\text{}$.

However, traditional Q-Learning struggles with large or continuous state spaces, as it requires storing and updating a Q-value for every possible state-action pair. This limitation makes it impractical for high-dimensional problems such as scan sequence optimization in LPBF. To overcome this challenge, DQL replaces the Q-value table with a deep neural network, known as a deep Q-network (DQN), that approximates the Q-value function. By leveraging deep learning, DQL can efficiently handle large, complex state spaces and is well-suited for sophisticated decision-making tasks.

In this paper, DQL was employed to efficiently explore the vast search space for scan sequence optimization. In this framework, the heat source in LPBF (e.g., a laser or electron beam) serves as the agent; the action corresponds to choosing the next scan location in the sequence, and the environment is represented by a physics-based simulator for LPBF. The action space A includes all possible scan locations within a layer, such as individual lines in a stripe pattern or islands in a chessboard pattern. For each selected action, the simulator generates the resulting temperature field. The reward function is specifically designed to reflect spatial thermal uniformity, guiding the agent toward optimal scan strategies that improve temperature distribution.

**Fig. 2:**
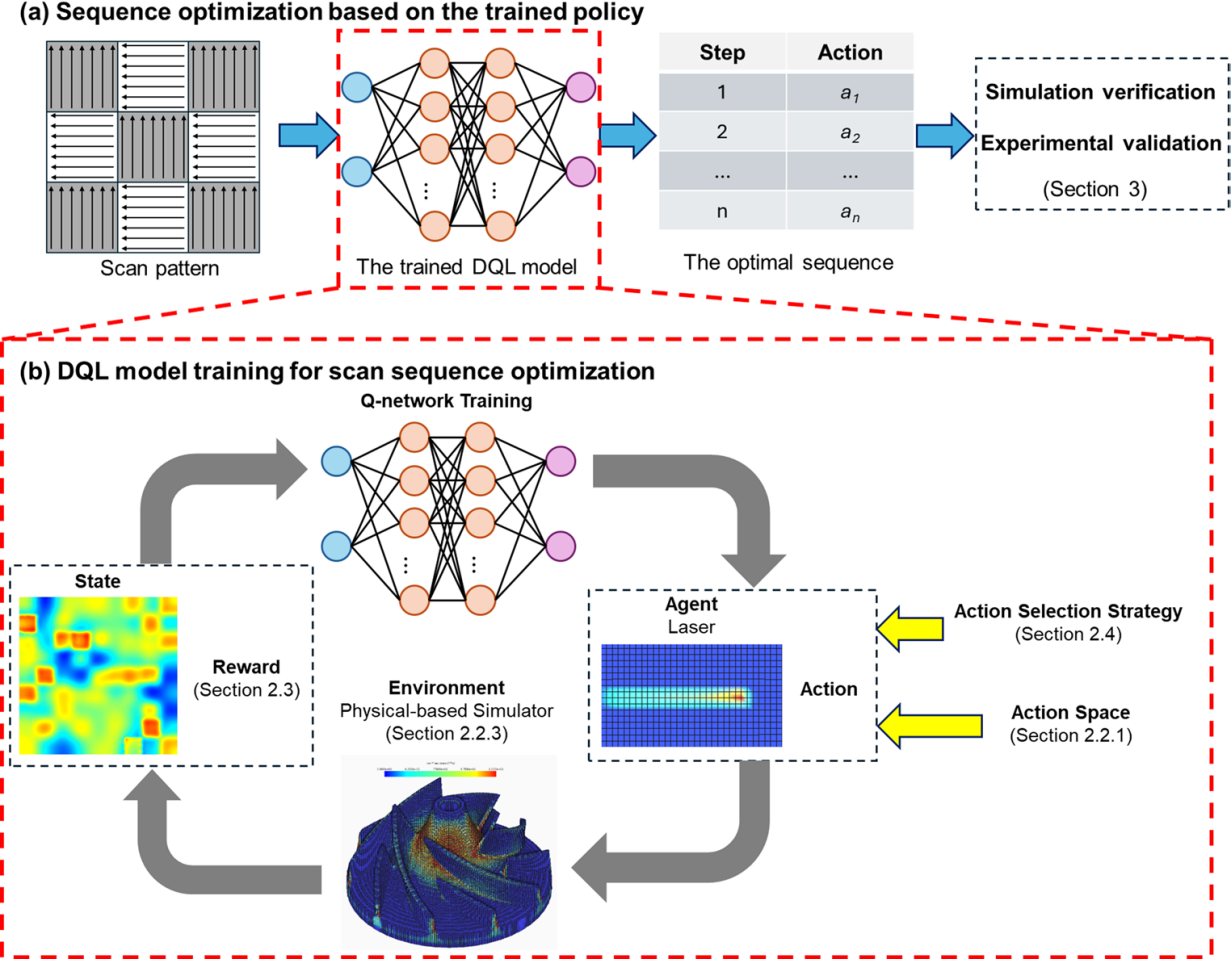
The complete workflow of the DQL-based algorithm for scan sequence optimization: (**a**) generation of optimized scan sequence using the trained DQL model, (**b**) DQL model training process

Through DQL, the agent learns to select the next scan location to enhance temperature uniformity. The complete workflow of the DQL-based Reinforced Scan Optimization framework is illustrated in Fig. 2, where Fig. 2**(a)** shows the generated optimal scan sequence and Fig. 2**(b)** depicts the trained action-selection policy. The DQL model is trained to derive this policy by iteratively updating the DQN over multiple episodes until convergence. Convergence indicates that the model has stabilized and consistently selects actions that maximize the cumulative reward, signifying that the agent has learned an effective and near-optimal scanning strategy.

The Reinforced Scan optimization method uses a structured DQL framework to generate optimal scan sequences in LPBF. It begins by defining the scan pattern and action space (Sect. 2.2.1), dividing the printing area into manageable regions to enable hierarchical optimization (Sect. 2.2.2). The DQL agent, representing the heat source, selects the next scan location based on the current temperature field, balancing exploration and exploitation (Sect. 2.4) while interacting with a physics-based simulator (Sect. 2.2.3). A custom reward function (Sect. 2.3) evaluates thermal uniformity, guiding the agent to minimize residual stress. The system transitions to a new state after each action, continuing this learning cycle through multiple episodes until convergence. Optimized sequences from all hierarchical levels are then combined (Sect. 2.2.2) to construct a globally optimized scan path. The entire process is summarized in Algorithm 1.

**Figure:**
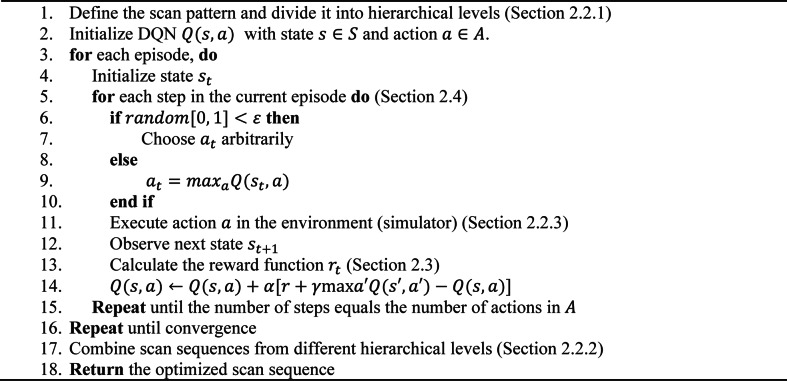
Algorithm 1: DQL-based algorithm for scan sequence optimization

### Action space design and evaluation of actions

#### Action space design

In the Reinforced Scan, the design of action space ***A*** is another important aspect, as it defines the set of all possible actions $\:{a}_{i}$ that the agent can take during optimization. The action space defines the set of all possible actions that the RL agent can take at any given state.

2 $${\boldsymbol A}_{\mathbf i}=\left\{a_j\right|j=1,\:2,\:3\dots\:n\}\;\left(i=1,2,...\right)$$

where *i* denotes the hierarchical level and $\:{a}_{j}$ represents the actions from 1 to *n* in the corresponding hierarchical level. Each hierarchical level is managed by a separate DQL model that operates at a different resolution of the scan pattern (details in Sect. 2.2.2). For example, the first level consists of larger blocks of the scan pattern, while the second level focuses on lines within these blocks. At the first level, the action space $\:{\boldsymbol{A}}_{1}$ comprises all possible locations of the blocks. Each action at this level corresponds to selecting the next block to be scanned. At the second level, the action space $\:{\boldsymbol{A}}_{2}$ includes all possible locations of the individual lines within a block. Each action at this level corresponds to selecting the next line to be scanned within a block.

#### Hierarchical scan sequence determination

**Fig. 3:**
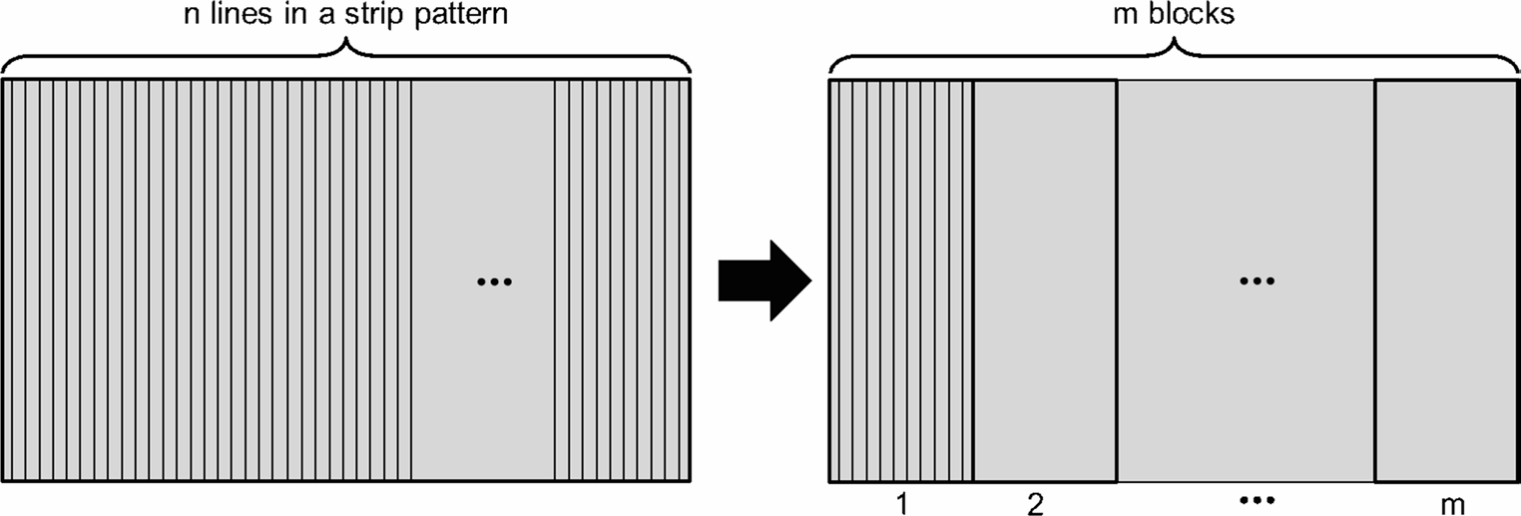
Hierarchical optimization levels for scan sequence in LPBF: Stripe pattern divided into blocks and lines

Hierarchical optimization is a key component of the Reinforced Scan framework, designed to efficiently address the complexity and computational demands of scan sequence optimization in LPBF. This approach decomposes the optimization problem into multiple hierarchical levels, with each level handled by an independent DQL model, thereby significantly reducing the overall search space. The process begins by dividing the scan pattern into smaller, more manageable segments, as illustrated in Fig. 3. The figure demonstrates how a stripe pattern can be partitioned into blocks and individual scan lines. This hierarchical structure enables scan sequence optimization at multiple spatial scales, ensuring a more comprehensive and effective optimization across the entire scan area.

To construct the final global scan sequence, the optimized block-level and intra-block line-level sequences are systematically combined through an interleaving approach. Let the optimized block sequence be represented as [*b₁*,* b₂*,* b₃*,* …*,* bₙ*], where each *bi* denotes a block to be scanned in the order determined by the first-level DQL model. Similarly, let the optimized line sequence within each block be represented as [*l₁*,* l₂*,* l₃*,* …*,* lₖ*], where each *lⱼ* denotes the relative scanning order of lines within a block, as determined by the second-level DQL model. The final scan sequence is generated by iterating over the positions in the intra-block line sequence and applying them across all blocks following the optimized block order. In other words, the scanning begins with line *l₁* in each block in the order [*b₁(l₁)*,* b₂(l₁)*,* b₃(l₁)*,* …*,* bₙ(l₁)*], then proceeds to line *l₂* in the same manner: [*b₁(l₂)*,* b₂(l₂)*,* …*,* bₙ(l₂)*], and so on until all lines *l₁* through *lₖ* are covered in every block. This interleaved execution across blocks, guided by their optimized sequence, enables the scan path to distribute thermal energy more uniformly over the build area at each stage.

#### Evaluation of actions using physics-based simulation model for temperature distribution

The temperature distribution during the LPBF process is governed by the heat conduction equation, which describes how heat is transferred within the material during laser scanning:

3 $$\:\rho\:{c}_{p}\frac{\partial\:T}{\partial\:t}=\nabla\:\bullet\:(k\nabla\:T)+Q$$

where 𝑇 is the temperature, 𝑡 is time, 𝜌 is the material density, $\:{c}_{p}$ is the specific heat capacity, 𝑘 is the thermal conductivity, and 𝑄 is the volumetric heat input, representing the laser energy source. This equation describes the fundamental physics of heat transfer in the LPBF process. The left-hand side of the equation represents the rate of change of temperature over time, while the first term on the right-hand side, $\:\nabla\:\bullet\:(k\nabla\:T)$, represents heat diffusion, which governs how heat spreads from high-temperature regions to the surrounding material. The second term on the right-hand side, 𝑄, represents the energy input from the laser, which melts the powder and generates a moving heat source as the laser scans across the build area. Together, this equation enables the prediction of thermal distributions resulting from different scan strategies.

In this paper, a finite element method (FEM) simulator developed in MATLAB™ is employed to solve the heat equation for different scan sequences. The simulator evaluates the temperature distribution resulting from the interaction between the laser and the material under various scanning paths. The output temperature field serves as an essential evaluation for calculating rewards in the Reinforced Scan framework.

### Reward function design

The objective of the reward function is to accurately represent the thermal uniformity of the scan area, enabling the DQL algorithm to optimize the scan sequence in order to reduce residual stress. Residual stress in LPBF primarily originates from non-uniform temperature distributions. Regions experiencing steep temperature gradients or rapid cooling contract unevenly, producing localized plastic deformation and accumulated residual stress after solidification [3, 8]. Therefore, reducing the temperature non-uniformity is critical for mitigating residual stress in LPBF parts.

**Fig. 4:**
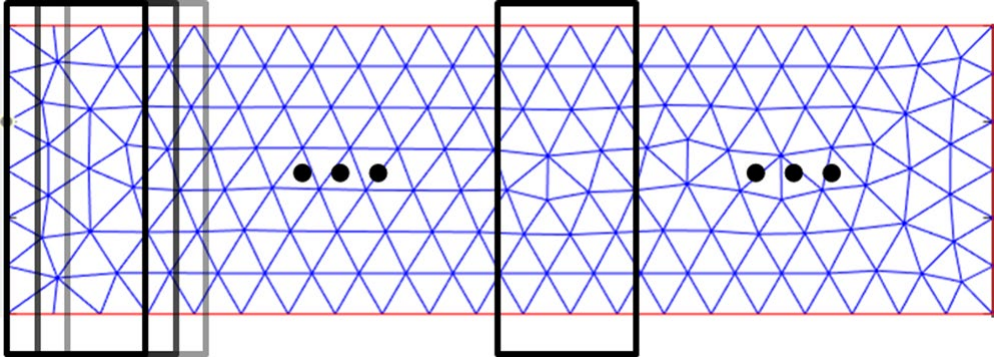
An example of the predefined window moving through the simulation area

Commonly used reward functions in the literature, such as temperature variance and energy density [10, 19], exhibit notable limitations. Temperature variance captures only the numerical dispersion of temperature values and fails to account for spatial uniformity, which is essential for assessing temperature gradients. Conversely, energy density overlooks the dynamic nature of heat transfer over time. Therefore, to effectively minimize residual stress, a more comprehensive reward function is required. In this work, we propose a reward function composed of three components: temperature variance ($\:{\sigma\:}^{2}\left(t\right)$), temperature discrepancy (TD), and high-temperature cluster distance (TCD):

4 $$\:r={-\sigma\:}^{2}\left(t\right)+TD+TCD-P$$

where 𝑃 represents the penalty for selecting previously visited actions, used to enforce the constraint that each location in the LPBF process must be scanned exactly once. In DQL-based optimization, this means that no action should be missed or repeated during the learning process. To ensure compliance with this requirement, a large penalty value is assigned whenever the agent attempts to select an action that has already been executed. This discourages redundant scans and guides the agent to explore new scan locations, thereby maintaining the validity of the scan path. The three components, namely, temperature variance ($\:{\sigma\:}^{2}\left(t\right)$), temperature discrepancy (TD), and high-temperature cluster distance (TCD), are calculated based on the output of the physics-based simulator. Prior to computing the rewards, the temperature values at each node in the simulation are normalized to a scale between 0 and 1. The temperature variance is then computed using the following equation:

5 $$\:{\sigma\:}^{2}\left(t\right)=\frac{1}{n}{\sum\:}_{i=1}^{n}{\left({T}_{i}-{T}_{avg}\right)}^{2}$$

where *n* is the total number of nodes in the mesh, *T*_*i*_ represents the temperature reading for each node, and *T*_*avg*_ is the average temperature for all nodes. Temperature variance measures the deviation of individual temperatures from the average, providing a general sense of thermal dispersion. However, since it does not account for the spatial arrangement of nodes, it lacks the ability to capture spatial thermal uniformity. To address this limitation, two additional metrics—temperature discrepancy and high-temperature cluster distance—are introduced. Temperature discrepancy quantifies the deviation in average temperatures across different regions of the scan area. In the FEM simulation, temperature discrepancy (TD) is defined as follows:

6 $$\:TD=\frac{{T}_{min}}{{T}_{max}}$$

7 $$\:{T}_{min}=Min\left({T}_{i}^{W}\right)$$

8 $$\:{T}_{max}=Max\left({T}_{i}^{W}\right)$$

where *T*_*min*_ and *T*_*max*_ are the minimum and maximum window temperatures, respectively, while $\:{T}_{i}^{W}$ denotes the average temperature for window *i*. The window is a subset of the simulation nodes, as shown in Fig. 4. A predefined window will move a uniform distance to iterate through the entire simulation area. For each movement, the average temperature of the nodes within the window is calculated and recorded as $\:{T}_{i}^{W}$. After the iteration, the *T*_*min*_ is defined by the lowest temperature among all recorded average temperatures and *T*_*max*_ is defined by the highest temperature among all recorded average temperatures. As TD describes the temperature ratio between the low and high-temperature areas, when TD is low, it means the high and low-temperature nodes are more gathered in a small area, and thermal uniformity is low. While the *T*_*min*_ is close to *T*_*max*_ (TD is close to one), the average temperatures in different locations are similar to each other, which indicates good uniformity for the temperature field.

**Fig. 5:**
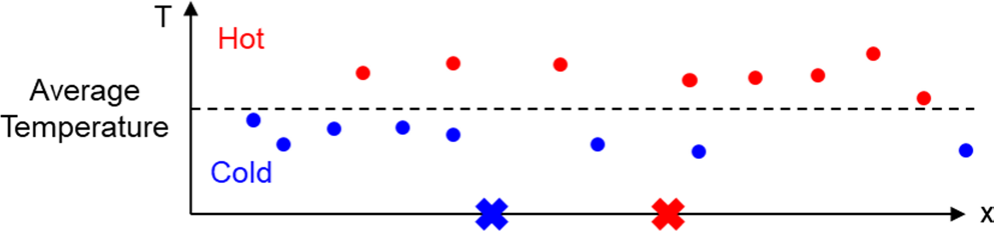
Illustration of TCD: red and blue dots represent nodes above and below the average temperature. $\:{N}_{H\_center}$ and $\:{N}_{C\_center}$​ are virtual nodes describing the average positions of hot and cold clusters, represented by red and blue crosses, respectively. TCD measures the dispersion of high-temperature nodes, with larger distances indicating better thermal uniformity

The TD identifies locations with peak and low temperatures; however, it is not a complete description of spatial thermal uniformity since it only considers the highest and lowest window temperatures. To address this limitation, the high temperature cluster distance (TCD) is used as an additional measure to describe global thermal uniformity, as shown in Fig. 5. It is defined as:

9 $$\:TCD=\raisebox{1ex}{$\sum\:_{i=1}^{\left|{\boldsymbol{N}}_{\boldsymbol{h}\boldsymbol{o}\boldsymbol{t}}\right|}d\left({\boldsymbol{N}}_{\boldsymbol{h}\boldsymbol{o}\boldsymbol{t}}\right(i),{N}_{H\_center})$}\!\left/\:\!\raisebox{-1ex}{$l$}\right.$$

10 $$\:{\boldsymbol{N}}_{\boldsymbol{h}\boldsymbol{o}\boldsymbol{t}}=\{x\in\:\boldsymbol{N}|{T}_{x}>{T}_{mean}\}$$

11 $$\:{N}_{H\_center}=\frac{\sum\:_{i=1}^{\left|{\boldsymbol{N}}_{\boldsymbol{h}\boldsymbol{o}\boldsymbol{t}}\right|}{\boldsymbol{N}}_{\boldsymbol{h}\boldsymbol{o}\boldsymbol{t}}\left(i\right)}{\left|{\boldsymbol{N}}_{\boldsymbol{h}\boldsymbol{o}\boldsymbol{t}}\right|}$$

12 $$\:{T}_{mean}=\frac{\sum\:_{i=1}^{\left|\boldsymbol{N}\right|}{T}_{i}}{\left|\boldsymbol{N}\right|}$$

where the $\:{\boldsymbol{N}}_{\boldsymbol{h}\boldsymbol{o}\boldsymbol{t}}$ is the subset of all nodes in simulation ***N*** with temperatures higher than the average temperature. The $\:{N}_{H\_center}$ is a virtual node whose coordinates are the average of all nodes in $\:{\boldsymbol{N}}_{\boldsymbol{h}\boldsymbol{o}\boldsymbol{t}}$. $\:{T}_{mean}$ is the average temperature of all nodes. $\:l$ represents the scale factor of the scan area. In this study, it is defined by the length of the longest side of the scan area. The TCD measures how dispersed the high-temperature nodes are, with a larger TCD indicating better thermal uniformity. A tightly clustered group of hot nodes implies localized heat accumulation, steep local temperature gradients, and consequently high localized residual stress. A larger TCD indicates that hot regions are more spatially dispersed, reducing local thermal concentration and lowering the likelihood of forming high-gradient residual-stress zones.

With the temperature variance ($\:{\sigma\:}^{2}\left(t\right)$), temperature discrepancy (*TD*), and high temperature cluster distance (*TCD*), the proposed reward function can effectively represent the thermal uniformity of the printing area, providing a solid foundation for optimization.

### Exploration and exploitation trade-off

The epsilon greedy approach is widely used in RL to balance exploration and exploitation in model training [28]. This approach compares a randomly generated value with epsilon$\:\left(\epsilon\:\right)$, a variable with its value between 0 and 1. If the $\:\epsilon\:$ is larger, the action with greater Q value will be chosen; otherwise, a random action will be picked. To balance exploration and exploitation, $\:\epsilon\:$ is defined in Eq. 12 [28].

13 $$\:\epsilon\:={\epsilon\:}_{f}\text{}+({\epsilon\:}_{0}-{\epsilon\:}_{f}\text{})\cdot\:\mathrm{e}\mathrm{x}\mathrm{p}(-m/\lambda\:)$$

where $\:{\epsilon\:}_{0}$​ is the initial epsilon value, $\:{\epsilon\:}_{f}$ is the final epsilon value, *m* is the current episode number, and $\:\lambda\:$ is the epsilon decay coefficient. The epsilon value decreases as the number of episodes increases, starting from $\:{\epsilon\:}_{0}$​ and decreasing exponentially towards $\:{\epsilon\:}_{f}$​. By adjusting the epsilon decay coefficient $\:\lambda\:$, the epsilon decreasing rate can be controlled: a lower $\:\lambda\:$ results in a faster decrease, resulting in less exploitation, while a higher $\:\lambda\:$ slows down the decrease, allowing for adequate exploration. The epsilon greedy approach ensures that the algorithm explores more at the beginning and gradually shifts towards more exploitation of the learned policy as training progresses.

## Case study

To further introduce the proposed Reinforced Scan optimization method, two case studies are conducted in this section: (1) a stripe pattern (Sect. 3.1) and (2) a chessboard pattern (Sect. 3.2), which are two commonly applied printing patterns in LPBF. Reinforced Scan interacts with the physics-based simulator (Sect. 2.2.3) to gradually optimize the scan sequence for better thermal uniformity. The objective of this optimization is to improve the uniformity of the thermal distribution during printing and further minimize the residual stress after printing. To demonstrate the effectiveness of Reinforced Scan, it is compared with three benchmark methods that are representative of commonly used scan strategies in LPBF: the Sequential scan sequence, the LHI method [14, 15], and SmartScan [10]. These methods were chosen because they reflect different optimization approaches. The Sequential method serves as a basic baseline without thermal consideration. LHI is a heuristic strategy that reduces thermal accumulation by maximizing the distance between consecutive scans. SmartScan is a thermal-model-driven approach that selects the next scan location based on minimizing local temperature variance. Numerical simulation verification of these four scan sequences is conducted using Netfabb™ Local Simulation. The average and maximum residual stress are compared. For the chessboard pattern, experiments are conducted on a customized laser LPBF platform to validate the optimization results.

### Case one: Stripe pattern optimization

In this case study, a stripe pattern scan sequence was optimized for LPBF. The objective is to achieve a more uniform temperature distribution, thereby minimizing residual stress in printed samples.

#### Simulation setup

**Fig. 6:**
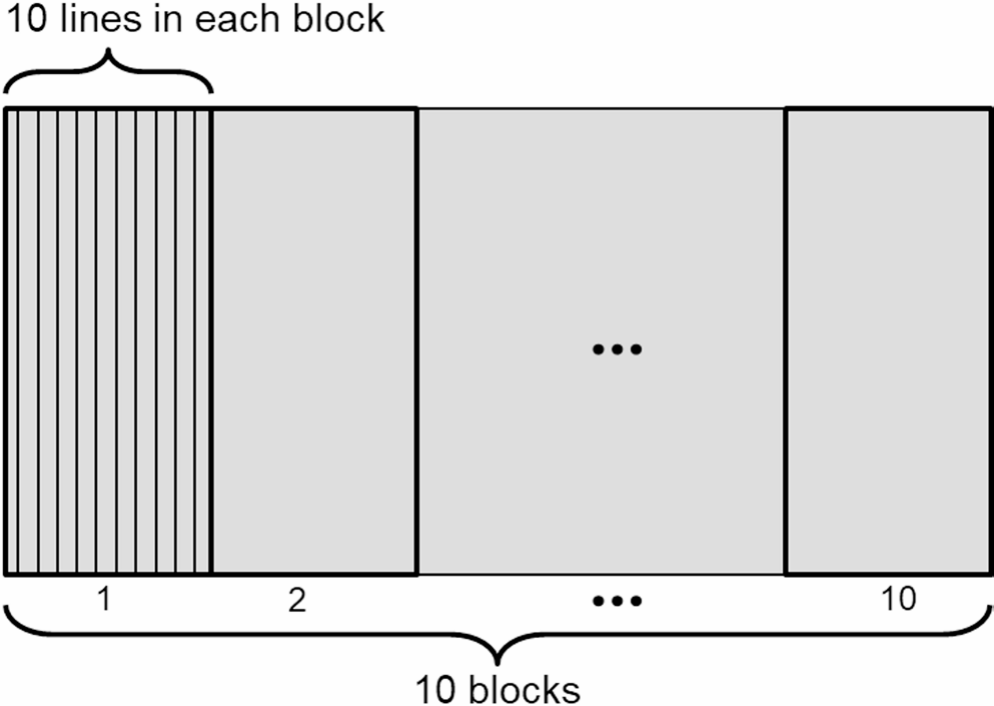
Illustration of scan pattern in case one: a 10 mm × 5 mm rectangular area with 100 scan lines, grouped into 10 blocks of 10 lines each

The scan pattern is a 10 mm × 5 mm rectangular area comprising 100 scan lines. As shown in Fig. 6, the scan pattern is divided into 10 blocks, each block consisting of 10 adjacent scan lines. This organization is based on the hierarchical scan approach introduced in Sect. 2.2.2. Both blocks and scan lines are numbered sequentially from left to right, from 1 to 10. The material used for the substrate is Ti-6Al-4V, a widely used titanium alloy in AM due to its excellent mechanical properties and corrosion resistance.

The physics-based thermal finite element simulator was developed to model the LPBF process, as introduced in Sect. 2.2.3. Process parameters were selected based on values reported in the literature and further refined through practical experience on our customized LPBF platform (introduced in Sect. 3.2.3) [29]. In this paper, the simulation applied a laser power of 200 W, a beam diameter of 120$\:\mu\:m$, and a hatch spacing of 100 $\:\mu\:m$. The scan-sequence optimization in this case study follows a two-level hierarchical structure. The first level is a global block-level optimization that partitions the scan area into blocks and determines the order in which these blocks are scanned. The second level is a fine-level optimization that determines the detailed sequence of individual scan lines within each block. The reinforcement learning model is trained independently at both levels, and the final scan path is obtained by combining the optimized block sequence with the optimized line sequence inside each block.

At the global level, each block consisting of ten scan lines is approximated as a single aggregated thermal unit. Under this assumption, all lines within a block are treated as being scanned within a short time interval, and the total energy input is represented using an effective heat source of 2000 W (10 × 200 W). This abstraction greatly reduces computational cost while still allowing the model to capture thermal variation across blocks. Once the optimal block order is obtained, the fine-level optimization is performed within each block using the standard laser parameters. This fine-level model accurately captures line overlap, remelt, and local temperature gradients, ensuring the necessary physical fidelity at the detailed level.

This type of approximation is consistent with approaches used in the thermal-modeling literature. For example, in references [30, 31], an entire layer was considered to be scanned at the same time, effectively treating it as a single large unit. While such models do not reproduce intra-layer temperature distribution precisely, they still offer valuable insight into thermal variability across layers. It enables the simulator to provide reasonably accurate temperature distributions for each scan sequence, which are then used to assess thermal uniformity and update the state in the DQL model training process.

#### Reinforced Scan implementation

Two DQL models were trained for this case study: one for determining the sequence of blocks, and another for determining the sequence of lines within each block. The first model, referred to as the Block Sequence Model, identifies the optimal scanning order for the 10 blocks. The second model, the Line Sequence Model, determines the optimal order for scanning the 10 lines within each block.

In terms of training details, the state is defined by the current temperature field of the scan pattern, while each action corresponds to selecting the next block or line to be scanned. The input to both neural networks consists of the temperature values at mesh nodes obtained from the physics-based simulation results (see Sect. 2.2.3). Each DQL model, at both the block and line levels, is composed of four fully connected layers, including two hidden layers with 2048 and 1024 neurons, respectively. The hidden layers use the Rectified Linear Unit (ReLU) as the activation function.

The action selection policy for the laser agent is based on the Q-values predicted by the output layer; the next scan location is selected by choosing the action with the highest Q-value. The primary training parameters and their respective values, listed in Table 1, were fine-tuned through an iterative trial-and-error process. The implementation was carried out in a Python environment, with the DQN models developed using the PyTorch framework. During training, both the epsilon parameter and the moving-averaged reward function curves were monitored.

**Table 1:** Training parameters for the DQL model

Training Parameters	Values
Learning rate ($\:\gamma\:$)	0.0001
Batch size	32
Discount factor	0.99
Initial epsilon ($\:{\epsilon\:}_{0}$)	1
Final epsilon ($\:{\epsilon\:}_{f}$)	0
Epsilon decay coefficient ($\:\lambda\:$)	500

#### Results

**Fig. 7:**
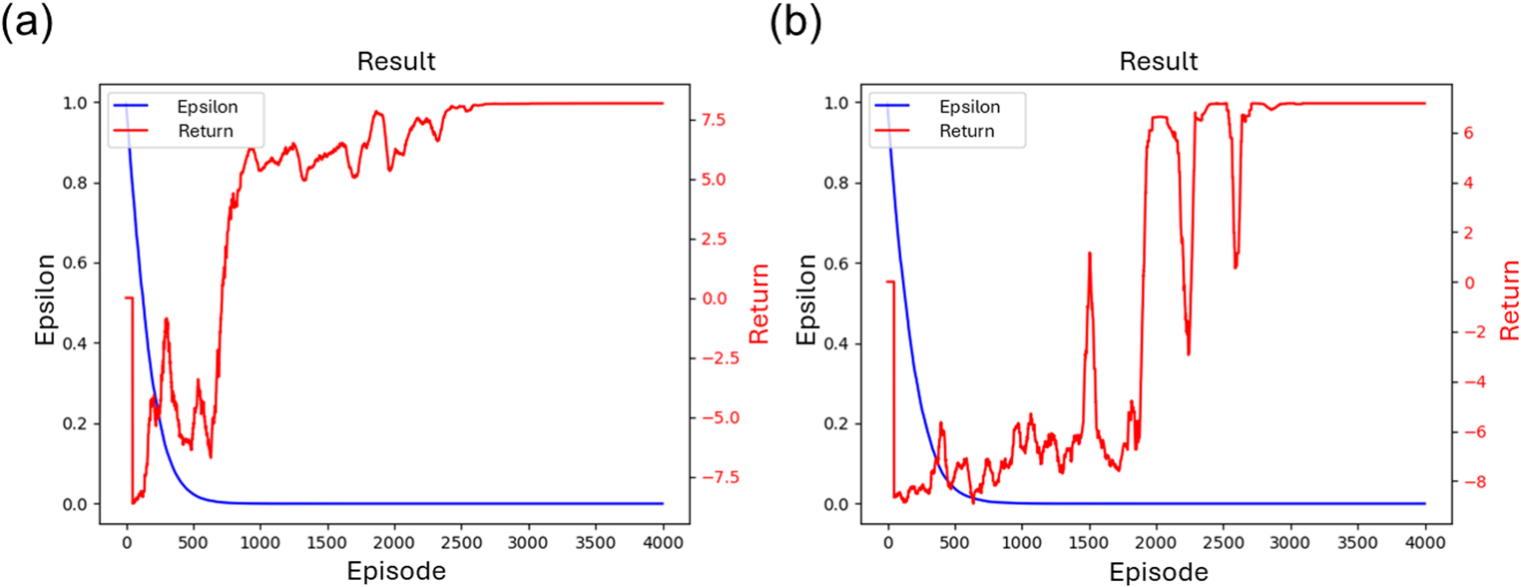
(**a**) Training process of the Block Sequence Model, (**b**) Training process of the Line Sequence Model

The training process of the DQN model for the two hierarchical levels is shown in Fig. 7. Figure 7(a) and (b) present the training progress for two DQL models. It indicates that the return (accumulated rewards, see Sect. 2.1) stabilized after around 3000 episodes, suggesting that the final scan sequence is consistent. The oscillations (peaks and valleys) in the return curves arise from the exploration–exploitation behavior. Early in training, frequent exploration produces alternating high and low returns as the agent tests sub-optimal and promising scan sequences. As epsilon decreases, exploration diminishes, the policy converges, and the return stabilizes at a higher level, indicating learning convergence.

**Fig. 8:**
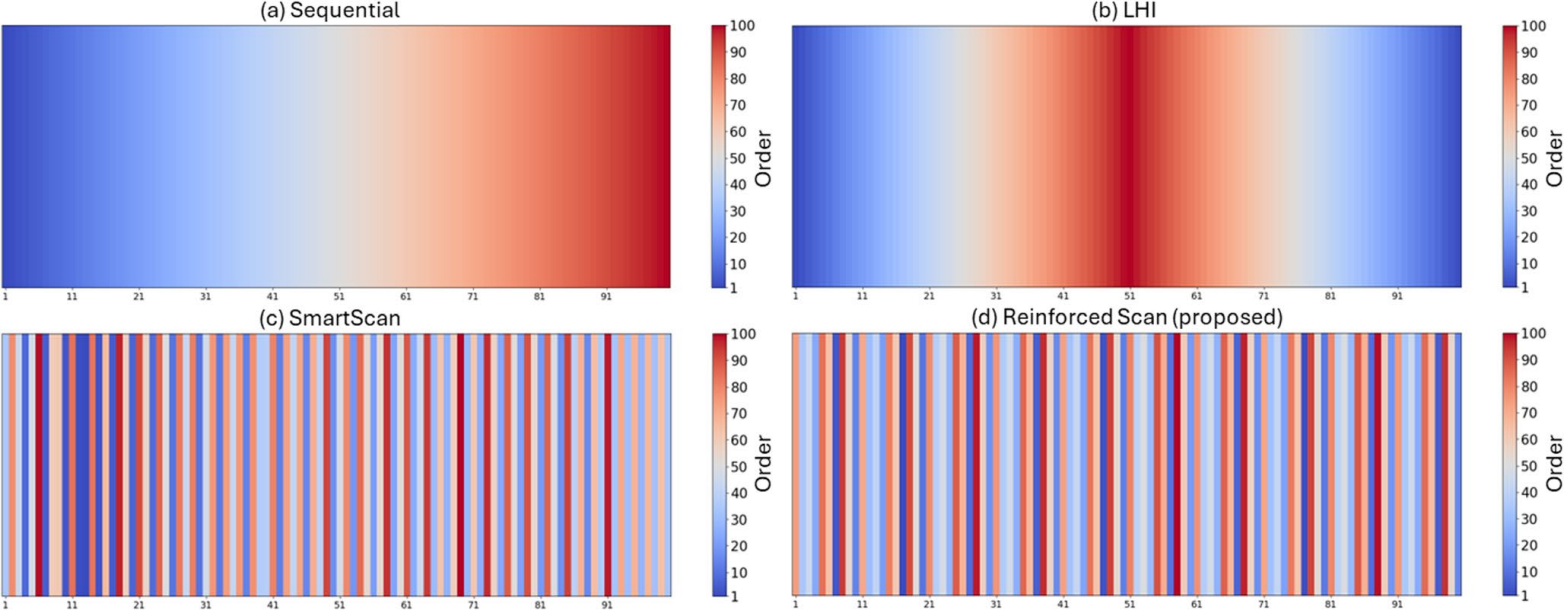
Color map of four scan sequences for the stripe pattern: (**a**) Sequential, (**b**) LHI, (**c**) SmartScan, and (**d**) Reinforced Scan (proposed)

To evaluate the effectiveness of Reinforced Scan, its optimized scan sequence was compared with three benchmark methods [7, 8][4], as shown in Fig.8. These methods were previously introduced as representative approaches (Sect. 3). The figure visualizes the scanning order using a color map, where blue indicates early scan lines and red represents later ones. In the Sequential scan sequence (Fig. 8(a)), the scan order progresses smoothly from one end to another, indicating that each line is scanned in direct succession. The LHI pattern (Fig. 8(b)) shows that the sequence begins near the edges and alternates inward toward the center. SmartScan (Fig. 8(c)) presents a highly irregular and seemingly random distribution of scanning order, with no clear directional pattern, indicating that the scan lines were selected based on localized thermal criteria. The Reinforced Scan (Fig. 8(d)) also shows a non-sequential, non-uniform distribution, but with a more balanced spread of early and late scan lines across the entire region.

The quality metrics considered in this study include the average and maximum residual stress of the single-layer post-printing sample, evaluated through Netfabb™ Local Simulation [32]. Netfabb™ Local Simulation is a widely adopted simulation tool for metal AM that provides thermo-mechanical modeling capabilities. It predicts temperature distribution, residual stress, and part deformation based on user-defined scan strategies. Its accuracy and compatibility with custom scan inputs make it well-suited for verifying scan path optimization methods in LPBF.

The printing parameters used in the Netfabb™ Local Simulation were selected to reflect typical LPBF conditions for Ti-6Al-4V and are aligned with those used in the earlier physics-based simulation (Sect. 3.1.1). Specifically, a laser power of 200 W, scan speed of 1200 mm/s, beam diameter of 120 $\:\mu\:m$, and hatch spacing of 100 $\:\mu\:m$ were applied to ensure consistent thermal modeling and reliable comparison across simulation environments. Table 2 summarizes the simulated residual stress results. The findings indicate that the Reinforced Scan method significantly outperforms the benchmark methods. Specifically, it achieved the lowest average residual stress at 309.18 MPa and the lowest maximum residual stress at 603.43 MPa. This represents a substantial improvement over the Sequential method, which exhibited the highest residual stresses—an average of 327.17 MPa and a maximum of 717.46 MPa. Compared with the SmartScan method, Reinforced Scan reduced the average residual stress by approximately 1.48% and the maximum residual stress by about 14.77% for the stripe pattern.

**Table 2:** Simulated residual stress for the Stripe pattern

	Sequential	LHI	SmartScan	Reinforced Scan (proposed)
Average residual stress (MPa)	327.17	329.42	313.81	309.18
Maximum residual stress (MPa)	717.46	605.33	708.00	603.43

**Fig. 9:**
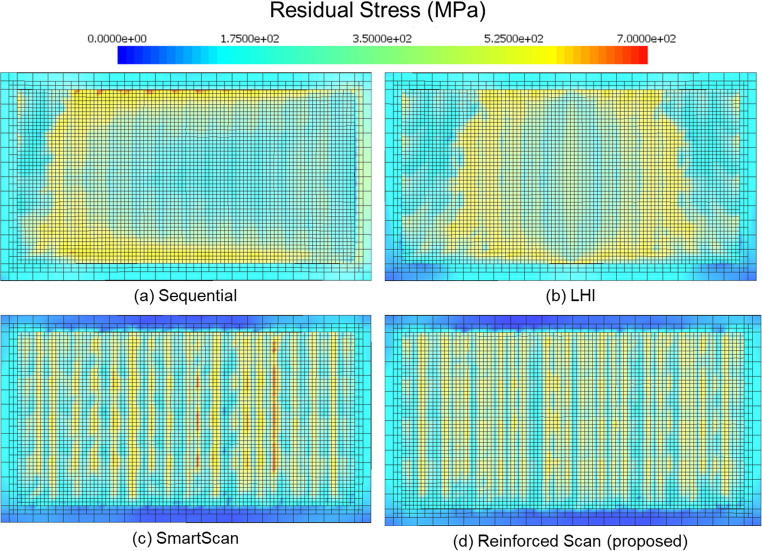
Residual stress distribution in the stripe pattern for four different scan sequences: (**a**) Sequential, (**b**) LHI, (**c**) SmartScan, and (**d**) Reinforced Scan (proposed)

Figure 9 shows the simulated residual stress distributions for four different scan sequences: (a) Sequential, (b) LHI, (c) SmartScan, and (d) Reinforced Scan. The color map represents residual stress levels in megapascals (MPa), with blue indicating low stress and red indicating high stress.

In the Sequential scan sequence (Fig. 9(a)), stress accumulates heavily along the outer edges of the printed layer. A broad region of moderate to high stress spans large area of the interior, suggesting a progressive buildup of thermal gradients as the scan progresses linearly across the layer. The LHI method (Fig. 9(b)) shows a different residual stress distribution pattern, where stress concentrations are symmetrically located near the center of the part. This is consistent with the alternating inward scan sequence used in LHI, which results in convergence of heat flow toward the middle of the build area. While edge stresses are slightly reduced compared to the Sequential method, central zone remains at a moderate stress level. In the SmartScan result (Fig. 9(c)), stress patterns become more uniform, forming vertical bands with alternating high and low stress. The distribution appears more spatially varied, and high-stress areas are thinner and less extensive than in the first two methods. This indicates some improvement in local thermal management, although stress variation remains high in some areas of the pattern. The Reinforced Scan (Fig. 9(d)) displays a notably more uniform residual stress field. High-stress regions are narrower and more evenly distributed across the scan area, with large portions of the layer dominated by low to moderate stress levels (green to light blue). There is minimal clustering of high residual stress values, and the pattern lacks the large continuing zones of elevated stress seen in the other methods. This visual evidence supports the conclusion that Reinforced Scan achieves a more balanced thermal distribution, leading to reduced residual stress.

### Case two: chessboard pattern optimization

In case two, the optimization of a chessboard scan pattern was conducted. As a widely adopted scan strategy in AM, this case study includes both simulation-based verification using Netfabb™ Local Simulation and experimental validation to demonstrate the effectiveness of the Reinforced Scan method.

#### Scan sequence optimization

**Fig. 10:**
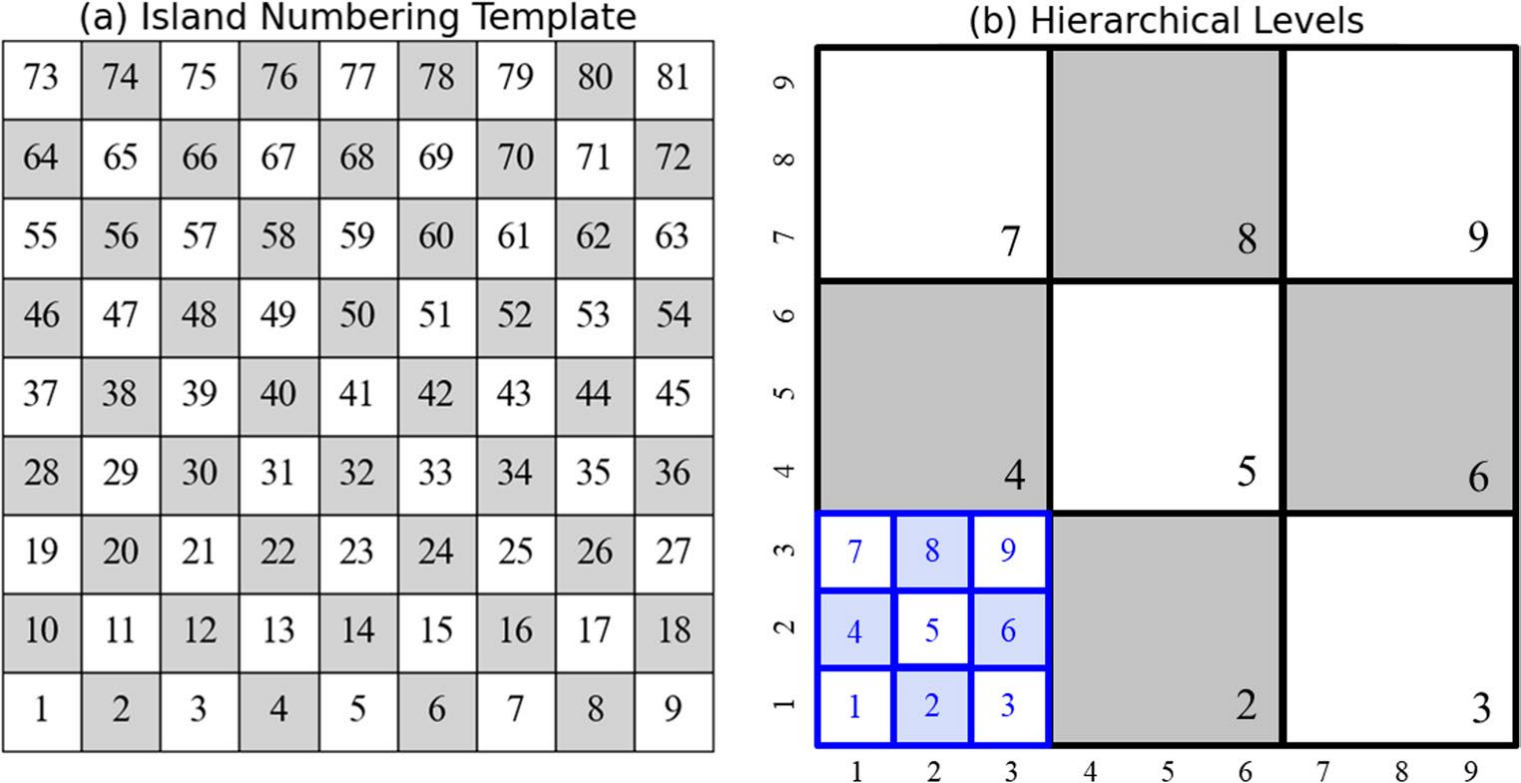
The hierarchical levels in the chessboard pattern: (**a**) islands numbering template, (**b**) hierarchical levels. The first-level clusters are shown in black boxes, while the second-level individual islands within these clusters are shown in blue boxes

The scan pattern is a 21.4 mm × 21.4 mm area, consisting of 81 individual islands. The material used for the substrate is Ti-6Al-4V. Eighty-one individual islands are grouped into 9 island clusters as the first hierarchical level, and the nine islands in each cluster construct the second level, as shown in Fig. 10. The simulator calculates the temperature distribution resulting from different scan sequences to evaluate thermal uniformity and update the state for DQL model training.

Same as case one, two separate DQL models were trained for this case study. The first model, the Island Cluster Sequence Model, determines the optimal order for scan clusters of islands in the chessboard pattern. The second model, the Individual Island Sequence Model, determines the optimal order for scanning the individual islands within each cluster.

**Fig. 11:**
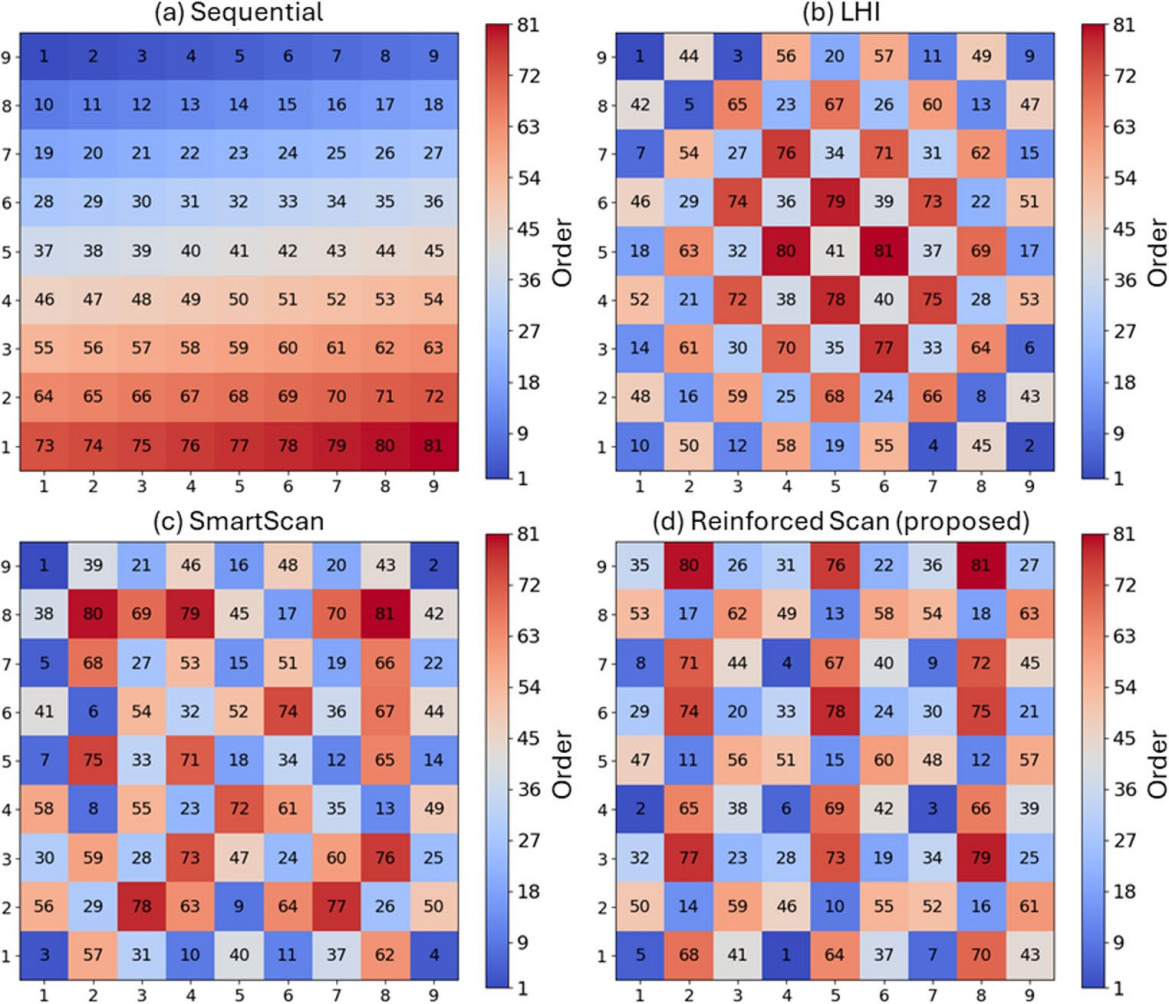
Color map of four scan sequences for the chessboard pattern: (**a**) Sequential, (**b**) LHI, (**c**) SmartScan, and (**d**) Reinforced Scan (proposed)

The architecture and input-output structure of the DQN are consistent with those used in the stripe pattern optimization case. The optimized scan sequences for the chessboard pattern are shown in Fig. 11, which compares four methods: (a) Sequential, (b) LHI, (c) SmartScan, and (d) Reinforced Scan. The figure uses a color map to indicate scan order, where blue represents early scan positions and red indicates later scan positions. In Fig. 11(a), the Sequential method follows a straightforward top-to-bottom order across rows, resulting in a smooth gradient of scan order from blue (early) at the top to red (late) at the bottom. Figure 11(b), corresponding to the LHI method, shows a near symmetric pattern where the scan begins from the edges and alternates inward toward the center. This distribution creates a cluster of late scans (in red) around the center islands. Figure 11 (c), SmartScan, exhibits a seemingly random scan order, with scattered early and late scans across the pattern without a clear spatial trend. Figure 11(d), the proposed Reinforced Scan, presents a more balanced scan order. Early and late scan indices are spread throughout the area, avoiding clustering and creating a spatially diverse pattern. This arrangement helps prevent thermal buildup in localized regions.

#### Simulation verification

To evaluate the effectiveness of the Reinforced Scan, the optimized scan sequence was compared with three other methods. The quality metrics assessed include the average and maximum residual stress of the printed sample, evaluated using Netfabb™ Local Simulation. The results demonstrate that the Reinforced Scan method significantly outperforms other benchmark methods. As shown in Table 3, it achieved the lowest average residual stress at 255.83 MPa and the lowest maximum residual stress at 700.65 MPa. Compared with the SmartScan method, the Reinforced Scan achieved an average residual stress reduction of approximately 3.90% and a maximum residual stress reduction of 1.91% in the chessboard pattern.

**Table 3:** Simulated residual stress for chessboard pattern

	Sequential	LHI	SmartScan	Reinforced Scan
Average residual stress (MPa)	362.69	281.21	266.22	255.83
Maximum residual stress (MPa)	731.75	719.72	714.32	700.65

**Fig. 12:**
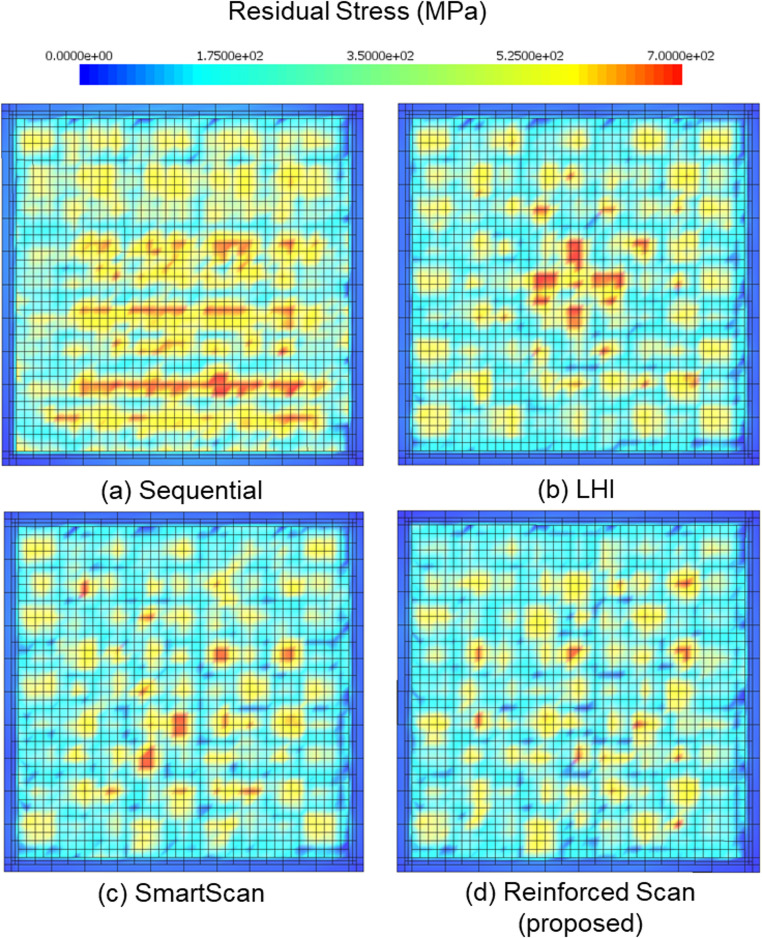
Residual stress distribution in the chessboard pattern for four different scan sequences: (**a**) Sequential, (**b**) LHI, (**c**) SmartScan, and (**d**) Reinforced Scan (proposed)

Figure 12 shows the simulated residual stress distributions for four scan strategies applied to the chessboard pattern: (a) Sequential, (b) LHI, (c) SmartScan, and (d) Reinforced Scan. Stress is represented in megapascals (MPa) using a color scale, where blue indicates low stress and red indicates high stress, with the upper bound reaching 700 MPa.

In Fig. 12(a), the Sequential scan shows a concentration of residual stress in horizontal bands toward the lower half of the layer. These high-stress regions are mostly aligned along rows, indicating the influence of the line-by-line scanning order. Edge stresses are moderate, but the lower interior region contains several broad red zones of high stress, showing that heat buildup persists in linearly adjacent scan regions. Figure 12(b), representing the LHI method, features a symmetrical stress pattern with a significant cross-shaped high-stress zone located in the center of the scan area. This pattern corresponds to the inward-focused scanning strategy of LHI, where overlapping thermal inputs converge near the middle. The surrounding regions display scattered medium stress areas, but the central accumulation is visually high and intense. In Fig. 12(c), the SmartScan method produces a more dispersed and irregular stress field. High-stress areas are fewer in number, smaller in size, and more evenly distributed throughout the printing area. Although some red spots remain, particularly in the central area, there is no dominant clustering of stress, suggesting improved thermal distribution. Figure 12(d), the proposed Reinforced Scan, displays the most uniform stress distribution among all four methods. High-stress points are minimal and isolated, with no noticeable patterns of clustering or banding. The stress is more evenly distributed over the entire layer, and the map is largely dominated by blue to light green tones, reflecting low to moderate stress levels. This pattern suggests that the scan sequence effectively minimized thermal gradients throughout the part and further mitigated the residual stress.

**Fig. 13:**
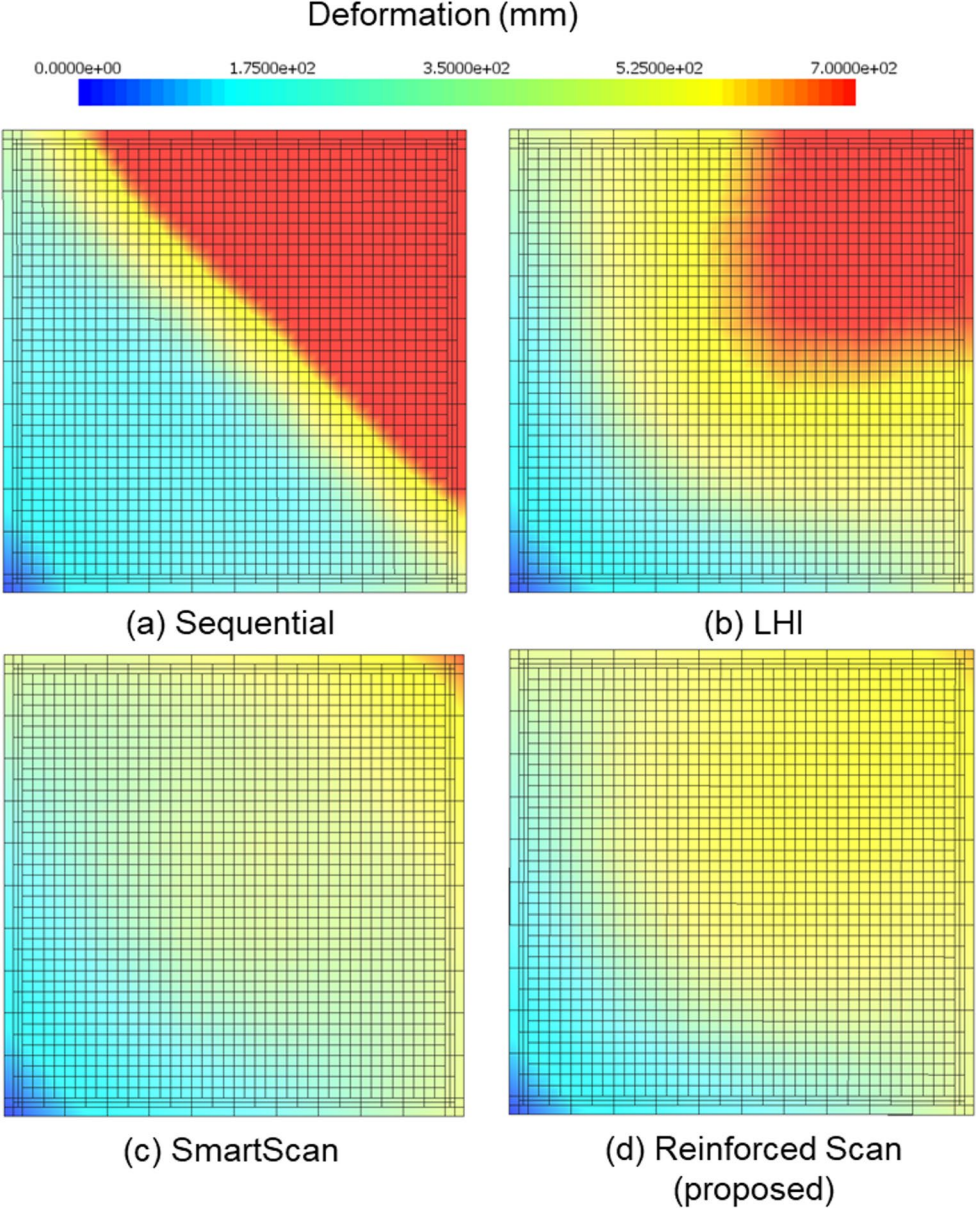
Deformation distribution in the chessboard pattern for four different scan sequences: (**a**) Sequential, (**b**) LHI, (**c**) SmartScan, and (**d**) Reinforced Scan (proposed)

In addition to residual stress distribution, the simulation also predicts the deformation for each scan strategy. Figure 13 shows the simulated deformation distributions of the printed Ti-6Al-4V thin plates for four scan sequences: (a) Sequential, (b) LHI, (c) SmartScan, and (d) Reinforced Scan. The deformation values are measured in millimeters and visualized using a color map, where blue indicates minimal displacement and red corresponds to maximum deformation.

In Fig. 13(a), the Sequential scan exhibits the highest deformation among all methods, with a maximum displacement of 0.167 mm. A large diagonal region across the top-left to bottom-right corner displays intense red and orange color, indicating strong warping concentrated in one half of the plate. This asymmetric pattern reflects unbalanced heat buildup due to the sequential scanning strategy. Figure 13(b), corresponding to the LHI method, shows an improved result with a maximum deformation of 0.052 mm. While the distribution is less extreme than in Sequential, a significant red region remains, and the gradient is still steep from corner to center. This suggests that although thermal accumulation is improved, it is not fully mitigated. Figure 13(c), the SmartScan method, further reduces deformation, with a maximum value of 0.036 mm. The deformation pattern is smoother and more evenly spread, with only a small localized region near the upper right showing notable displacement. The majority of the layer lies within the green-to-yellow range, suggesting moderate and well-controlled warping. Figure 13(d), the Reinforced Scan, achieves the best performance with a maximum deformation of only 0.035 mm. The deformation is minimal and uniformly distributed across the plate, with no high-intensity zones. This result visually and numerically confirms that Reinforced Scan successfully minimizes thermal distortion.

#### Experimental validation

Experimental validation was conducted for the chessboard pattern because it is widely adopted as a standard scan strategy in commercial LPBF systems and is thus more representative of practical industrial printing conditions. Unlike stripe patterns, the chessboard configuration introduces complex two-dimensional thermal interactions, including cyclic reheating between adjacent islands, localized heat accumulation, and island-boundary effects, all of which strongly influence residual stress and deformation behavior in LPBF parts [11, 12, 33–35]. These interactions create a more challenging and realistic thermal environment, making the chessboard pattern a stringent benchmark for evaluating the capability of the Reinforced Scan to manage spatial thermal non-uniformity. For this reason, the chessboard pattern was selected for full experimental verification to confirm the real-world applicability and performance of the proposed method.

**Fig. 14:**
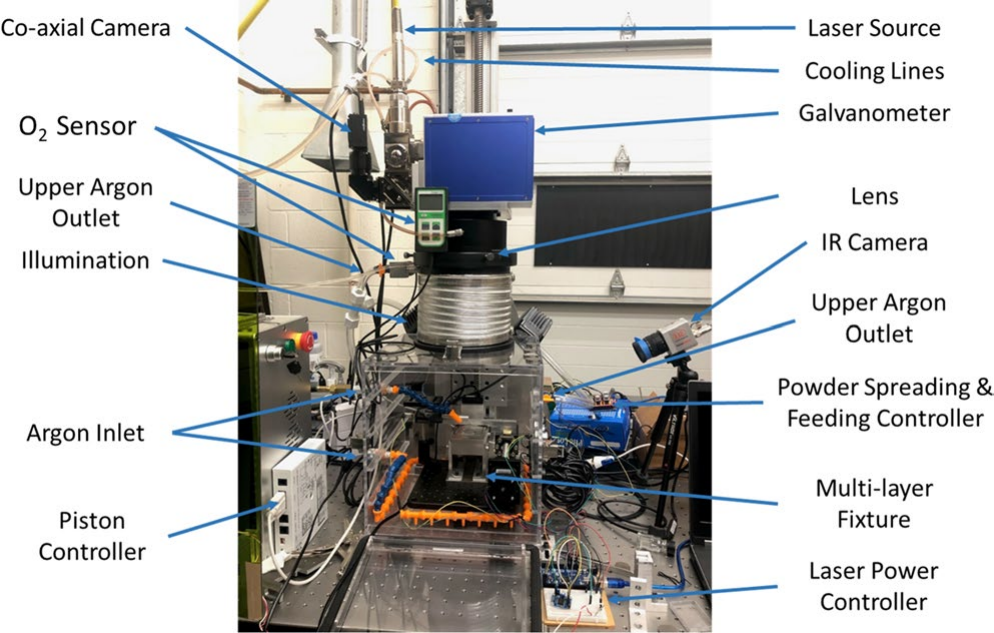
The customized LPBF platform used for experimental validation [29]

To validate the optimization results of the Reinforced Scan, experiments were conducted using a customized LPBF platform, as shown in Fig. 14. This platform is equipped with a 1000 W Raycus RFL-C1000 laser source, operating at a wavelength of 1080 ± 5 nm. It is paired with a PSH20HW galvo scanner from CARMAN HAAS and an F-theta lens.

In the experimental validation, the chessboard pattern was printed on a thin Ti-6Al-4V plate. The thin plates were 0.015 inch (0.38 mm) sliced by using a wire EDM machine. The same printing parameters used in the simulation were also applied in the experimental process. Four samples were printed using different scan sequences: traditional sequential scan, LHI method, SmartScan method, and the optimized scan sequence obtained from the Reinforced Scan. The primary quality metric for experimental validation was the deformation of the thin Ti-6Al-4V plate, a critical factor in evaluating the residual stress and effectiveness of the scan sequences [10, 19].

**Fig. 15:**
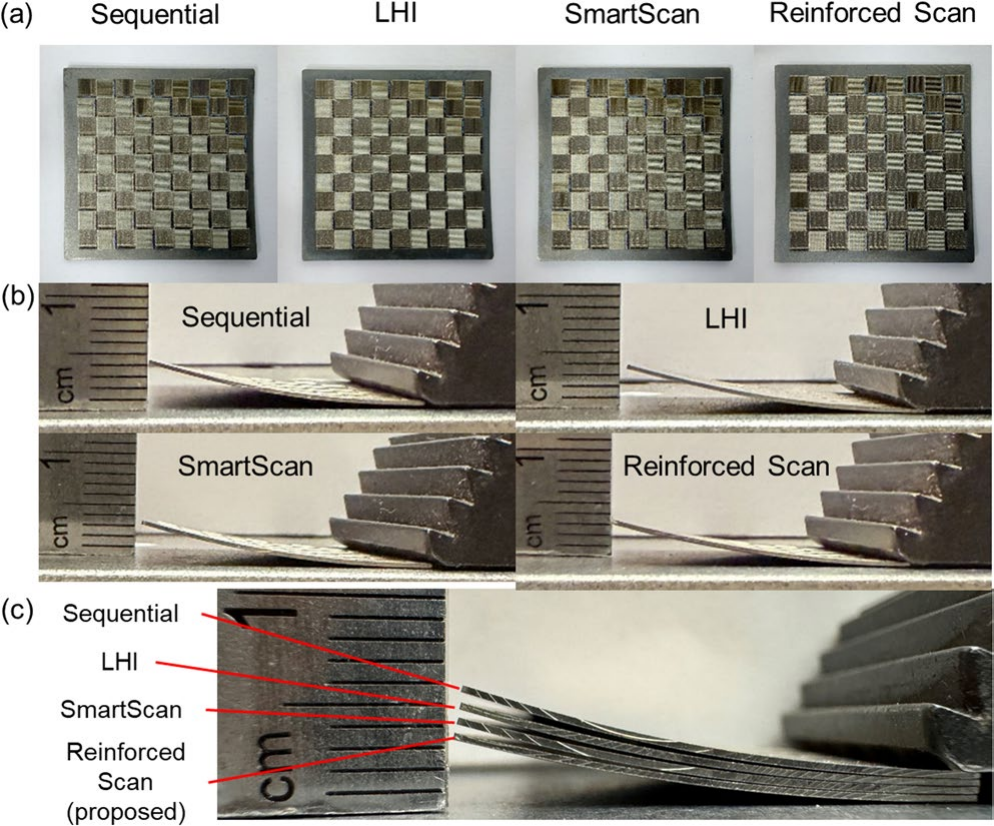
Experimental results for four different scan sequences. (**a**) Top view of the scanned area; (**b**) Side view of the scanned samples; (**c**) Stacked view of the four samples. The scan sequences are, from top to bottom in (**c**): Sequential, LHI, SmartScan, and Reinforced Scan (proposed)

Figure 15 shows the experimental results for the four Ti-6Al-4V samples with different scan strategies: Sequential, LHI, SmartScan, and Reinforced Scan. The top image shows the top view of the scanned chessboard patterns. The bottom image provides a side view of the samples, revealing the deformation caused by residual stress.

The deformation of the scanned chessboard patterns was measured individually using a digital caliper, and the maximum vertical displacement for each sample was recorded. The results align closely with simulation predictions. The sample scanned using the Sequential scan sequence exhibited the highest maximum deformation at 4.50 mm. This was followed by the LHI and SmartScan samples, which showed maximum deformations of 3.84 mm and 3.51 mm, respectively. The Reinforced Scan sample demonstrated the least deformation, measured at 3.21 mm, and appears visibly flatter than the others. This corresponds to an 8.55% reduction in deformation relative to SmartScan, indicating improved dimensional stability consistent with the simulation-based stress reduction trends. These results confirm the effectiveness of the Reinforced Scan in reducing residual stress and improving dimensional accuracy in LPBF-manufactured parts.

## Conclusion

This study proposes the Reinforced Scan, a reinforcement learning enabled scan strategy for optimizing scan sequences in PBF. The primary objective is to minimize residual stress in printed parts by achieving a more uniform temperature distribution during the printing process. The Reinforced Scan leverages the capabilities of DQL to explore the vast search space effectively. Its methodology involved dividing the scan pattern into hierarchical levels, with separate DQL models trained to optimize the sequence of different levels. The reward function was designed to reflect spatial thermal uniformity, guiding the agent towards an optimal scan sequence. In the case study, the simulation results show significant improvements for Reinforced Scan in residual stress compared with traditional methods. The experimental results closely aligned with simulation predictions, further confirming the effectiveness of the Reinforced Scan approach in reducing deformation in LPBF. Overall, this study provides an effective approach for efficiently optimizing the scan sequence in LPBF to reduce the residual stress in printed parts.
